# Kinetic Determination of Acetylsalicylic Acid Using a CdTe/AgInS_2_ Photoluminescence Probe and Different Chemometric Models

**DOI:** 10.3390/bios13040437

**Published:** 2023-03-30

**Authors:** Rafael C. Castro, Ricardo N. M. J. Páscoa, M. Lúcia M. F. S. Saraiva, João L. M. Santos, David S. M. Ribeiro

**Affiliations:** LAQV, REQUIMTE, Department of Chemical Sciences, Laboratory of Applied Chemistry, Faculty of Pharmacy, University of Porto, Rua de Jorge Viterbo Ferreira nº 228, 4050-313 Porto, Portugal

**Keywords:** quantum dots, acetylsalicylic acid, chemometrics, chemical analysis, second-order advantage

## Abstract

The combination of multiple quantum dots (QDs) in a multi-emitter nanoprobe can be envisaged as a promising sensing scheme, as it enables obtaining a collective response of individual emitters towards a given analyte and allows for achieving specific analyte-response profiles. The processing of these profiles using adequate chemometric methods empowers a more sensitive, reliable and selective determination of the target analyte. In this work, we developed a kinetic fluorometric method consisting of a dual CdTe/AgInS_2_ quantum dots photoluminescence probe for the determination of acetylsalicylic acid (ASA). The fluorometric response was acquired as second-order time-based excitation/emission matrices that were subsequently processed using chemometric methods seeking to assure the second-order advantage. The data obtained in this work are considered second-order data as they have a three-dimensional size, I × J × K (where I represents the samples’ number, J the fluorescence emission wavelength while K represents the time). In order to select the most adequate chemometric method regarding the obtained data structure, different chemometric models were tested, namely unfolded partial least squares (U-PLS), N-way partial least squares (N-PLS), multilayer feed-forward neural networks (MLF-NNs) and radial basis function neural networks (RBF-NNs).

## 1. Introduction

Quantum dots (QDs) have been extensively applied, in recent decades, as suitable nanoplatforms for chemical analysis since they can be used not only as reactive photoluminescent (PL) sensing elements but also as passive optical labels [1,2,3,4,5,6]. The typical utilisation of a single QD as a recognition element has, in recent years, progressed towards the combination of multiple QDs or the association of other types of luminophores with QDs in a combined multi-emitter nanoprobe, which, by assuring multiple measurements per analyte, can be envisaged as one of the most promising sensing schemes. Effectively, the cumulative response of all single emitter nanoprobes towards a specific chemical species allows for the obtaining of a specific analyte-response profile, which, after being analysed using adequate chemometric tools, empowers a more sensitive, reliable and selective determination of the target analyte [6,7,8,9].

As it is frequently highlighted in the scientific literature, the remarkable optical properties of semiconductor QDs make them particularly attractive to setup multicomponent reaction packages that are able to endure assorted magnitude reactions with the analyte, which therefore provide combined concentration-related fluorescent signals. Among these, the high molar attenuation coefficients and the broad excitation bands that allow the simultaneous excitation of several QDs of different sizes and/or compositions (different maximum emission wavelengths) by using a single excitation wavelength, should be emphasised. Their narrow emission bands are also relevant since they enable the combination of multiple fluorophores in the same probe without significant overlap of their emission bands [5,6,9]. Additionally, it is worth mentioning that the emission wavelength of these nanomaterials can be easily adjusted during the synthesis process, making it possible to obtain nanocrystals with different emission wavelengths covering the entire UV/visible-to-infrared electromagnetic spectrum [5,6,9].

Although convenient, in many circumstances the overlapping of the emission spectra could not be avoided. In fact, this hindrance restrains the number of QDs that could be used in the same assay and, commonly, a maximum of three spectrally resolved QD emitters could be combined without significant spectral overlap [6]. On the other hand, the higher the number of QDs used in the analysis, the more specific the response that is obtained, making the combined nanoprobe less susceptible to the interference of some species that might be present in the sample matrix. Conveniently, the use of mathematical and statistical methods, such as chemometric models, improves the acquisition of information through a PL data set and could easily circumvent possible selectivity issues. Additionally, these widely used methods could resolve the spectrum overlapping, allowing the combination of several fluorophores in the same probe [10,11].

Another common hindrance that could impair the chemical analysis of samples with complex matrices, which is one of the main challenges of analytical chemistry, is the quantification of analytes in the presence of unknown and uncalibrated interferents [6,11]. In this sense, the amount of acquired data, as well as the application of the most suitable chemometric model, are of utmost importance to carry out qualitative and/or quantitative analytical estimations with high selectivity [12,13,14,15]. Therefore, the use of second-order data combined with the appropriate chemometric model allows for the obtaining of the second-order advantage, which makes possible the determination of the target analyte, even in the presence of unknown and uncalibrated constituents of the sample matrix [16]. The data are classified as second-order or as a three-way array when a matrix per sample is obtained, such as the evolution of the PL spectrum throughout time (i.e., kinetic process of the interaction between QDs and analyte) [17,18] or when using an excitation–emission matrix (EEM) acquired via a rapid-scanning spectrofluorometer [19,20,21,22,23,24,25,26,27,28,29].

The combination of QDs as sensing platforms and a form of chemometric analysis for pharmaceutical applications has been explored as an alternative or complement to the official methods, which rely on chromatographic techniques that are usually time-consuming and laborious. Indeed, chemometric-assisted QDs-based PL methodologies can provide accurate, sensitive, selective and reliable results without requiring expensive equipment and skilled operators.

The most frequently used medicine worldwide is acetylsalicylic acid (ASA), commonly known as aspirin [30,31]. At high and intermediate doses, ASA is used as an anti-inflammatory, analgesic and antipyretic drug as it impairs the production of thromboxanes and prostaglandins through the inhibition of COX-1 and COX-2 enzymes [30,31,32,33,34]. At low concentration levels, ASA is used as an antithrombotic agent, and could be used to prevent cardiovascular disease (CVD). Moreover, recently, other therapeutic purposes have been investigated, namely the antitumor effect [35,36] or its impact on Alzheimer’s disease [37]. Additionally, ASA is frequently used in combination with other drugs in order to improve the therapeutic effect of the drugs [30]. Bearing this in mind, the development of analytical methodologies with enhanced selectivity for the determination of ASA in the presence of other active pharmaceutical ingredients (APIs) is of utmost importance. In addition, the monitoring of the amount of ASA in pharmaceutical formulations is essential to guarantee that the correct dosage of the medication is provided to the patient, a process aimed at achieving its intended therapeutic effects. The development of efficient analytical methodologies for ASA monitoring assures the safety and efficacy of the medication, contributing to the patient’s health and well-being.

In this work, a binary CdTe and ternary AgInS_2_ (AIS) QDs were combined in a PL nanoprobe for the determination of ASA in pharmaceutical formulations. The selected QDs exhibited emission bands with too-proximate maximum emission wavelengths; therefore, they were significantly overlapped, which, under normal circumstances, would impair the determination of ASA. However, to overcome this drawback, the kinetic interaction between the combined probe and the drug was thoroughly assessed in order to obtain three-way array data that were subsequently processed using appropriate chemometric models. As this kinetic interaction showed non-linear behaviour, different chemometric tools were tested and compared, namely unfolded partial least-squares (U-PLS), N-way partial least-squares (N-PLS), multilayer feed-forward neural networks (MLF-NNs) and radial basis function neural networks (RBF-NNs).

## 2. Materials and Methods

### 2.1. Chemicals and Solutions

All standards and solutions were prepared by employing chemicals of analytical grade without any purification or further treatment The solutions and standard were prepared using ultrapure water with a conductivity ≤ 0.1 μS cm^−1^ from a Milli-Q system.

A solution of 178 mg L^−1^ of acetylsalicylic acid intermediate was daily prepared by dissolving 17.8 mg of the acetylsalicylic acid standard reagent (C₉H₈O₄, Sigma-Aldrich^®^, ≥99%, St. Louis, MO, USA) in 100 mL of ultrapure water.

The CdTe QDs passivated with glutathione (GSH), 2-mercaptoethanesulfonate (MES), 3-mercaptopropionic acid (MPA) and thiomalic acid (TMA) were obtained by performing the microwave-assisted aqueous synthesis route proposed by Ribeiro et al. [38]. Likewise, the AIS QDs were prepared following the synthesis protocols as described in the scientific literature for TMA [39], MPA [40] and D-penicillamine (D-Pen) [40,41] capping agents. All distinctly capped CdTe QDs as well as the ternary D-Pen-capped AIS/ZnS QDs were precipitated with absolute ethanol (C_2_H_5_OH, 99.8%, Panreac, Barcelona, Spain) from the crude solution. Later, the precipitates were centrifuged, dried in a vacuum and kept in amber flasks, protected from light, for posterior use. Later, the intermediate solutions of QDs were obtained through the dissolution of the appropriate amount of the respective precipitates. For the remaining distinctly capped AIS QDs, the solution used in the analytical assays was prepared through the proper dilution of the crude solution obtained from the synthesis without any additional purification process.

Three distinct, commercially available pharmaceutical formulations containing 500 and 100 mg of ASA per tablet were used for the preparation of sample solutions. Two of these pharmaceutical formulations were a combination of two APIs, namely ASA/bisoprolol and ASA/caffeine. The sample solutions were treated by weighing an adequate amount of the powder in deionized water and shaking via mechanical stirring for 30 min to ensure an efficient drug extraction. Then, the solutions were filtered into a volumetric flask and the final volume was completed with deionized water.

### 2.2. Equipment

The CdTe and AIS QDs were synthesized in a CEM Discover SP^®^ Microwave system containing an automated pressure control/sensing system (ActiVent™), an active cooling system (PowerMAX™) and an integrated infrared (IR) sensor. A computer using Synergy™ software (Matthews, NC, USA) was used to monitor all the operations. The pH measurements were performed using a pH-meter GLP 22 (CRISON), and a Thermo Electron Jouan BR4I refrigerated centrifuge (Waltham, MA, USA) was employed for the separation of the precipitated QDs.

The PL data were collected using a Fluorolog^®^-3 spectrofluorometer (Horiba Jobin Yvon, NJ, USA), while the PL lifetime of all synthesized nanomaterials was measured by using a DeltaFlex^TM^ TCSPC lifetime spectrofluorometer (Horiba Scientific, Kyoto, Japan). Finally, the quantum yields (QYs) were recorded at room temperature with an Absolute QY Spectrometer (Quantaurus QY—C11347-11, Hamamatsu, Japan).

### 2.3. Fluorometric Assay and Sample Preparation

For the implementation of the kinetic measurements, the MES-CdTe intermediate solution was previously prepared by dissolving 4.9 mg of the powder in 6.5 mL of deionized water, obtaining a 0.75 mg mL^−1^ mass concentration. In the case of MPA-capped AIS QDs, a dilution in a ratio of 1:10 (*v*/*v*) of the crude solution was used (200 µL into a final volume of 2000 µL) with the aim of obtaining a similar fluorescence intensity regarding the binary QDs.

The assay involved, in the first stage, the addition of the required amounts of deionized water followed by the sequential addition of 100 µL of CdTe QDs and 50 µL of AIS QD intermediate solutions. Subsequently, proper amounts of ASA were added, giving a final volume of 2 mL. Immediately after the addition of the analyte, the resulting mixture was stirred, and the emission spectra were measured into a quartz cell (10 mm of optical path). The emission spectra were collected by fixing the excitation wavelength at 400 nm and by using slit widths of 5.0 nm for the excitation and emission. It was recorded every minute up to 10 min in an emission wavelength range from 460 to 800 nm.

### 2.4. Data Analysis

The obtained data in this work are considered as second-order data as they have a three-dimensional size, I × J × K (where I represents the samples number, J the fluorescence emission wavelength while K represents the time). Therefore, it is important to apply the most appropriate chemometric model. In this work, different chemometric models, namely U-PLS, N-PLS, MLF-NN and RBF-NN [42,43,44,45], were tested for the quantification of ASA. More information regarding these chemometric models can be found in the abovementioned literature.

For U-PLS and N-PLS, the optimal number of latent variables was estimated through leave-one-out cross validation and following Haaland and Thomas criterion [46]. This criterion basically states that the optimal number of latent variables is obtained when the prediction error sum of squares (PRESSs), which indicates how well a particular PLS model fits the concentration, is not statistically different (F-ratio probability below 0.75) to the minimum PRESS value obtained from models using a higher number of latent variables. Different pre-processing techniques, namely the standard normal variate and Savitzky–Golay filter methods with a different polynomial order or filter points, using either the first or the second derivative, were tested individually and in all possible combinations. After this optimization involving only the calibration samples, the optimized models were evaluated in terms of root-mean-square error of calibration (RMSEC) and the coefficient of determination for calibration (R^2^_C_). Then, the validation samples were projected into the best optimized models to evaluate the accuracy of the models and to obtain the figures of merit according to the equations given by Allegrini et al. [47]. The evaluation of the accuracy of the validation models was performed through root-mean-square error of prediction (RMSEP), coefficient of determination for the prediction (R^2^_P_), relative percentage error for concentration predictions (REs), limit-of-determination (LOD) and limit-of-quantification (LOQ).

All data analysis was performed using Matlab R2014 version 8.3 (MathWorks, Natick, USA) and the MVC2 graphical user interface [48].

Both MLF-NN and RBF-NN were performed through MVC1-GUI, which is a graphical user interface running under a Matlab (R2014 version 8.3) environment [49]. Before running the MVC1 graphical user interface, the original data were reduced through unfolded principal component analysis (U-PCA) using the MVC2 interface. In this process, the original data were mean-centred and a total of three components were selected. The architecture of both the NN models tested consists of three following layers: an input layer, a hidden layer and one output layer. The optimization of both NN models was performed under the back-propagation method and using only the calibration samples for testing different input and hidden layers neurons. This was conducted by trial and error until the training and monitoring errors stopped improving [43]. For the MLF-NN, the leaning rate, momentum and epoch parameters were set as 0.5, 0.5 and 2000, respectively, during the optimization, and the calibration data were further divided into 70% for training and 30% for monitoring. For the RBF-NN, the calibration data were not divided in training and monitoring sets as these models are tuned using only the calibration set through the minimization of a parameter called penalized cross-validation, which is similar to the root-mean-square error of calibration [50]. The learning rate, momentum and epoch parameters were not adjusted as this model is trained by least-squares and the weights are estimated automatically using the calibration set after the centres and widths of the Gaussian functions are estimated [50]. Again, different pre-processing techniques, namely the standard normal variate and Savitzky–Golay filter methods with different polynomial order and filter points, using the first and second derivative, were tested individually and in all possible combinations. After this optimization involving only calibration samples, the optimized models were evaluated in terms of RMSEC and R^2^_C_. Subsequently, the validation samples were projected into the best optimized models to evaluate the accuracy through RMSEP, R^2^_P_, RE, LOD and LOQ. More details about the calculation of these figures of merit can be found in [49]. The best architecture was found using 2 input layer neurons, 1/2/3 hidden layer neurons (depending on the pre-processing technique used) and 1 single output neuron.

The calibration set was composed of 13 standard solutions of ASA at concentrations ranging between 5.3 and 40.0 mg/L. The validation of the U-PLS, N-PLS, MLF-NN and RBF-NN models was performed using 3 commercial samples, each one using three different dilutions.

The emission fluorescence spectra were comprised between 460 and 800 every minute during the first 10 min. Therefore, each spectra contained a total of 341 × 11 = 3751 data points.

All data were mean-centred before the application of the abovementioned chemometric models.

## 3. Results and Discussion

### 3.1. Optical Properties of the QDs

All the synthesised QDs (TMA, GSH, MPA and MES-capped CdTe as well as D-Pen and TMA-capped AIS/ZnS and MPA and TMA-stabilized AIS) were characterized through time-resolved and steady-state fluorimetry.

**Figure 1:**
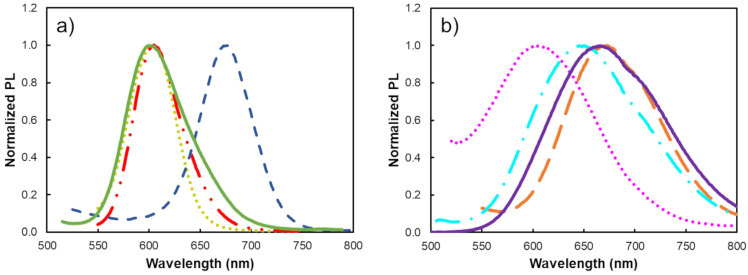
Normalized photoluminescence (PL) spectra of the (**a**) binary CdTe capped with thiomalic acid (TMA) (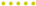), glutathione (GSH) (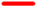), 3-mercaptopropionic acid (MPA) (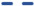) and 2-mercaptoethanesulfonate MES (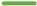) and (**b**) ternary AgInS_2_ quantum dots stabilized with MPA (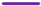) and TMA (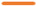) as well as AgInS_2_/ZnS quantum dots capped with D-penicillamine (D-Pen) (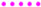) and TMA (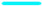).

The emission spectra of the synthesised nanomaterials showed that AIS QDs had broad emission bands, while the CdTe QDs displayed symmetric and narrower emission bands (Figure 1). This means that CdTe QDs sizes were more homogeneous and monodisperse than the ternary Cd-free ones. In fact, in the case of the ternary QDs, the characteristic emission band depends on distinct relaxation pathways which occur through electron–hole recombination and the radiative recombination of donor/acceptor mid-gap energy states within the band gap [18,39,51,52].

The PL lifetime mean values, the QY and the maximum emission wavelength of all synthesized QDs are summarized in Table 1 and Table 2. According to the obtained results, the PL decays (Figure S1—Supplementary Material) were appropriately fitted with two or three exponential kinetics. This fact demonstrates that the PL emission of the synthesised nanomaterials occurs through two or three different radiative processes. The obtained average lifetime was calculated following the equation described by Sillen et al. [53].

Through the comparison of the obtained results, one can conclude that the PL lifetime values significantly depend on the nature of the surface capping ligand and the nanomaterial composition and arrangement (CdTe or AgInS_2_ core, with and without ZnS shell). As expected, the PL lifetimes of binary CdTe QDs were approximately 10 times lower regarding those obtained with ternary AIS QDs. A possible explanation for this fact is that the ternary AgInS_2_ QDs have a more complex band structure than the binary CdTe QDs due to the presence of a third element in the core which results in additional intermediate energy levels within the band gap (mid-gap energy states) and, consequently, leads to a larger number of available radiative pathways for electron–hole recombination. In other words, the more complex band structure of ternary QDs increases the probability of radiative recombination and can lead to a longer photoluminescence lifetime.

In addition, PL lifetimes values also varied depending on the used capping ligand. The passivation of CdTe with MES showed lower PL lifetimes relatively to the nanoparticles capped with TMA, MPA and GSH. In the case of the ternary QDs, the passivation with D-Pen exhibited lower PL lifetime values. Since these capping ligands act as a passivation layer on the QDs’ surface, they prevent the recombination of photo-generated charge carriers and enhance their stability. So, different capping ligands can have distinct affinities for the QDs’ surface, affecting their ability to passivate the surface and consequently the PL lifetime.

### 3.2. Preliminary Assays

#### 3.2.1. Reactivity Assays

Aiming at selecting the most appropriate PL nanoprobe to determine ASA in pharmaceutical products, the reactivity of eight distinct QDs was assessed. As described in the scientific literature, the reactivity of QDs can be influenced by their nature (semiconductor or carbon dots), composition (CdTe or CdS, CuInS_2_ or AgInS_2_), size and arrangements (binary, ternary or quaternary) as well as by the nature of the passivating agent, which may contain distinct terminal functional groups (-NH_2_, -COOH, -SO_3_^−^, etc.). Effectively, these terminal functional groups not only determine the surface charge of nanoparticles but also confer the selectivity and sensitivity of the QDs towards a given analyte.

**Figure 2:**
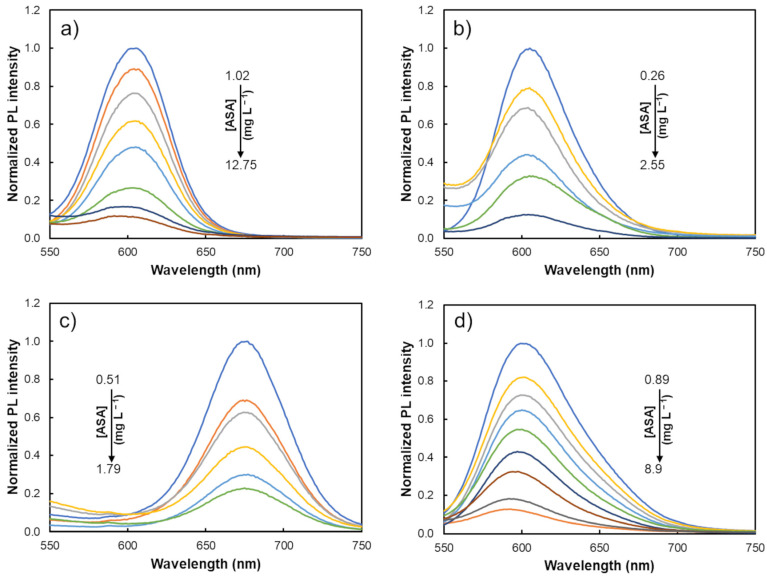
Normalized PL spectra of the nanoparticles sensing platforms: (**a**) TMA-CdTe QDs in the absence and presence of increasing concentrations of acetylsalicylic acid ASA ranging from 1.02 to 12.75 mg L^−1^; (**b**) GSH-CdTe alone and by adding increasing ASA concentrations between 0.26 and 2.55 mg L^−1^; (**c**) MPA-CdTe QDs before and after the interaction process with ASA at a concentration level from 0.51 to 1.79 mg L^−1^ and (**d**) MES-CdTe QDs alone and upon interacting with increasing concentrations of ASA (0.89–8.9 mg L^−1^).

**Figure 3:**
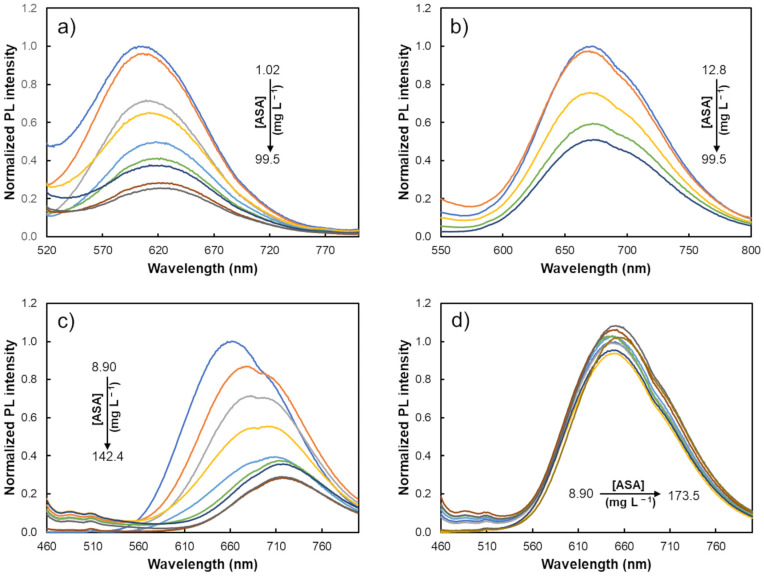
Normalized photoluminescence spectra of (**a**) D-Pen AIS/ZnS in the absence and presence of increasing concentrations of ASA ranging from 1.02 to 99.5 mg L^−1^; (**b**) TMA-AIS alone and by adding increasing ASA concentrations between 12.8 and 99.5 mg L^−1^; (**c**) MPA-AIS before and after the interaction process with ASA at a concentration level from 8.90 to 142.4 mg L^−1^ and (**d**) TMA- AIS/ZnS alone and upon interacting with increasing concentrations of ASA (8.90–173.5 mg L^−1^).

For this study, the concentration of all as-prepared PL nanomaterials was adjusted to a pre-determined value in order to ensure a similar fluorescence intensity. The final concentrations of the powder-stored TMA-, GSH-, MPA- and MES-CdTe QDs were 3.20, 1.83., 1.50 and 19.00 µg mL^−1^ (*w*/*v*), respectively, while for ternary D-Pen-capped AIS/ZnS QDs it was 20.00 µg mL^−1^ (*w*/*v*). For the others solution-stored ternary QDs, dilution factors of 1:10, 1:400 and 1:100 were used for TMA-AIS/ZnS, MPA-AIS and TMA-AIS QDs, respectively. The PL spectra of each nanomaterial in the absence and presence of increasing concentrations of ASA were acquired and depicted in Figure 2 and Figure 3.

The results revealed that upon the interaction of TMA-, GSH-, MPA- and MES-CdTe QDs with ASA, the PL properties of each nanocrystal were progressively quenched in an ASA concentration-dependent manner. Additionally, a slight blueshift in the maximum emission wavelength was observed, in particular in the cases of TMA-CdTe (605 nm to 591 nm) and MES-CdTe QDs (599 nm to 592 nm), thus demonstrating a different reactivity regarding the other distinctly capped binary QDs. The use of QDs with this kind of capping ligand, namely TMA or MES, can confer an improved selectivity for ASA determination. This hypsochromic effect can be possibly attributed to a slight reduction in QD size caused by capping removal upon oxidation at the QDs’ surface [54].

Concerning the ternary Cd-free QDs, the interaction study with ASA also demonstrated a progressive inhibition of the PL properties by increasing the aspirin concentration. Alongside the PL quenching effect, in the case of MPA-AIS QDs the interaction with the drug also showed a redshift in the maximum emission wavelength of up to 53 nm (665 to 718 nm). This bathochromic effect in the maximum emission wavelength of QDs can be explained by the increase in the nanoparticle size upon the interaction with the target analyte or by the formation of shallow-trap radiative recombination centres [55].

Additionally, in the case of the interaction between TMA-AIS/ZnS QDs and aspirin (Figure 3d), no significant variation in the PL intensity was observed. Effectively, the addition of a semiconductor shell material at the QDs’ surface allows not only to enhance the optical properties of the nanocrystals, improving the quality of the crystal lattice and protecting the core from being oxidized, but also to reduce its reactivity towards a given analyte, since the optical properties of the nanocrystals are less susceptible to changes in the medium or to the presence of other chemical species. In this case, the functionalization of the QDs’ surface with ZnS allows for the preservation of their optical properties when interacting with ASA, contrarily to what was observed by using TMA-capped AIS QDs without a ZnS shell.

#### 3.2.2. Stability Assays

**Figure 4:**
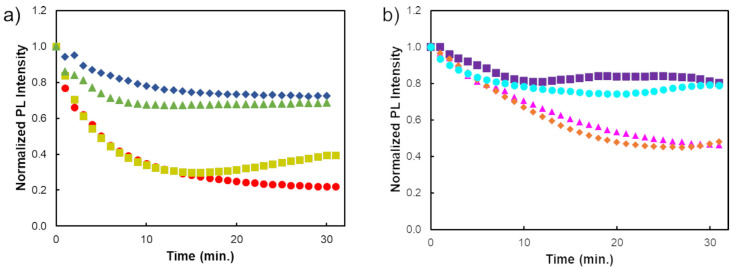
Evolution of the maximum PL intensity throughout 30 min of (**a**) binary CdTe QDs capped with GSH (●), MES (▲), TMA (■) and MPA (♦) and (**b**) ternary AIS QDs stabilized with MPA (■) and TMA (♦) as well as AIS/ZnS QDs capped with D-Pen (▲) and TMA (●).

The kinetic behaviour of each nanomaterial was evaluated not only to obtain second-order data but also to avoid the presence of possible interfering species in the pharmaceutical formulations through the second-order advantage. In some pharmaceutical formulations, ASA is not the only active pharmaceutical ingredient (API) present in the formulation. Therefore, to prevent the interference of unwanted species that could be present in the pharmaceutical formulations, second-order instrumental data were used. These allowed for a more accurate estimation of ASA concentration through the exploitation of the second-order advantage. The maximum emission intensity of the distinct binary and ternary QDs was evaluated in the absence of the analyte for 30 min; the obtained results are depicted in Figure 4.

The analysis of the results showed that the PL intensity of MES and MPA-capped CdTe QDs slightly diminished during the 30 min (∼31% and ∼27, respectively), while for TMA and GSH-capped CdTe QDs, a noticeable inhibition of the PL emission (∼70 and ∼72%, respectively) was observed up to 15 min, remaining constant for longer periods of time. Regarding Cd-free ternary QDs, the PL intensity of D-Pen-AIS/ZnS and TMA-AIS QDs decreases similarly for approximately 40% of the initial intensity. On the other hand, MPA-AIS and TMA-AIS/ZnS were the more stable ones, since their PL intensity only decreased by about 20% at the end of 30 min.

Considering the best compromise between the reactivity and stability of QDs, CdTe QDs stabilized with MES and AIS QDs passivated with MPA were chosen for the following assays. In fact, these nanomaterials reveal not only an accentuated stability throughout the 30 min but also an additional selectivity towards ASA due to the modification of their characteristic maximum emission wavelengths (a bathochromic shift in MPA-AIS QDs and hypsochromic effect in MES-CdTe QDs) upon the interaction with the drug.

#### 3.2.3. PL Quenching Mechanism of MPA-AgInS_2_ QDs and MES-CdTe QDs in the Presence of ASA

**Figure 5:**
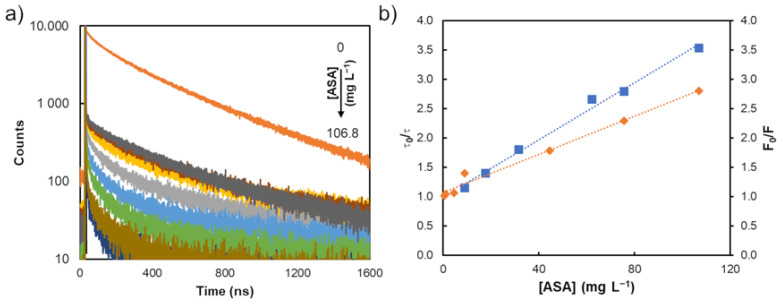
(**a**) PL decay curves of the MPA-AIS QDs (λ_em_ = 665 nm) in the absence and presence of increasing concentration of ASA. (**b**) Stern–Volmer plots displaying PL lifetimes ratio (orange diamonds) and the ratio of the PL intensities (blue squares).

The PL quenching mechanisms of the two quantum dots upon interacting with the aspirin were assessed by recording the PL lifetime decay values of the QDs both alone (τ_0_) and by adding increasing concentrations (τ) of ASA (up to 106.8 mg L^−1^ for MPA-AIS and up to 8.9 mg L^−1^ for MES-CdTe). The obtained PL lifetime decay values of MPA-AIS QDs decreased in the presence of increasing concentrations of ASA (Figure 5a). This inhibition can be described through the following equation:

$$\frac{\tau_{0}}{\tau }=0.0162\left(\pm 0.0009\right)\left[\mathrm{ASA}\right]+1.07\left(\pm 0.05\right)\;R^{2}=0.984,\;n=7$$

According to the observed, the slope of the Stern–Volmer plot obtained by analysing the PL intensities ratio (0.024 ± 0.001) was higher than the one observed by plotting the PL lifetimes ratio (0.0162 ± 0.0009). Effectively, in this interaction process, the inhibition of the PL intensity of the nanoparticles can be ascribed both to dynamic processes relying on nanoparticle/analyte collisions and to static processes through the formation of a complex with ASA (quencher). As the $\frac{F_{0}}{F}$ > $\frac{\tau_{0}}{\tau }$, it was possible to infer that both dynamic and static processes can occur [56]. Regarding the static process, and considering the high affinity between Ag^+^ and ASA, one can conclude that a complex between the drug and the metal ion was formed at the ternary QD’s surface [57]. On the other hand, the most probable dynamic process which contributes to the QDs PL quenching mechanism was a charge transfer process, since the ASA’s absorption band and Cd-free QDs’ emission bands were not significantly overlapped. Effectively, when dynamic processes occurred, the collisional interactions between the quencher and the excited QDs depopulated the excited state without PL emission upon non-radiative transitions to the ground state, and consequently a decrease in the mean decay lifetime was verified [18,56].

**Figure 6:**
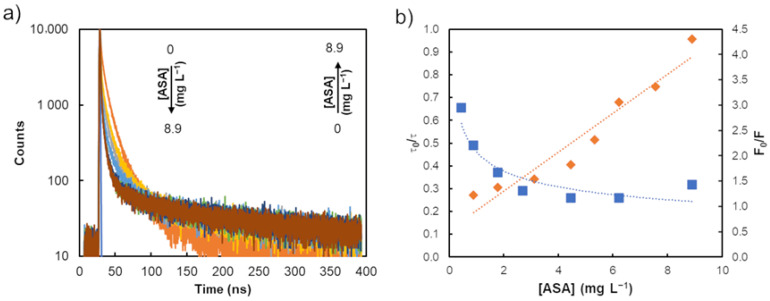
(**a**) Time-resolved PL decay curves of the MES-CdTe QDs (λ_em_ = 599 nm) in the presence of increasing concentration of aspirin. (**b**) Ratio of PL lifetimes (blue squares) and PL intensity (orange diamonds).

In the case of MES-CdTe, the time-resolved PL decay curves showed that with increasing concentrations of the drug, the PL lifetime decay values increased (Figure 6a).

### 3.3. Development of a CdTe/AgInS_2_ Photoluminescence Probe

Taking into account the different reactivities and stability of the tested nanoparticles, the combination of binary and ternary QDs could allow for the obtaining of a characteristic PL-response profile for ASA. Therefore, as mentioned above, MES-CdTe and MPA-AIS QDs were selected for the following assays.

As previously noted, despite the PL intensity of both QDs being inhibited in the presence of increasing concentrations of ASA, they exhibited dissimilar sensitivities for the analyte. In fact, for MES-CdTe QDs, a total PL quenching was observed after adding 8.9 mg L^−1^ of aspirin, whilst the total PL quenching of MPA-AIS QDs was only verified upon the addition of 142.4 mg L^−1^. Additionally, the higher sensitivity of binary MES-CdTe QDs towards the analyte was also observed when comparing the slopes of the linear Stern–Volmer plots that describe the PL responses obtained from both QDs/ASA interaction processes. Effectively, in the case of MPA-AIS QDs, a Stern–Volmer quenching constant (K_sv_) of 0.0162 (±0.0009) L mg^−1^ was obtained, while for MES-CdTe QDs, the K_sv_ was 23.5 times higher: 0.38 (± 0.04) L mg^−1^. In addition to the distinct reactivities demonstrated by the two QDs, a specific PL-response profile could be obtained because, upon the interaction with ASA, a redshift in the maximum emission wavelength was observed for the ternary QDs while a slight blueshift of the binary QDs’ emission bands were verified.

**Figure 7:**
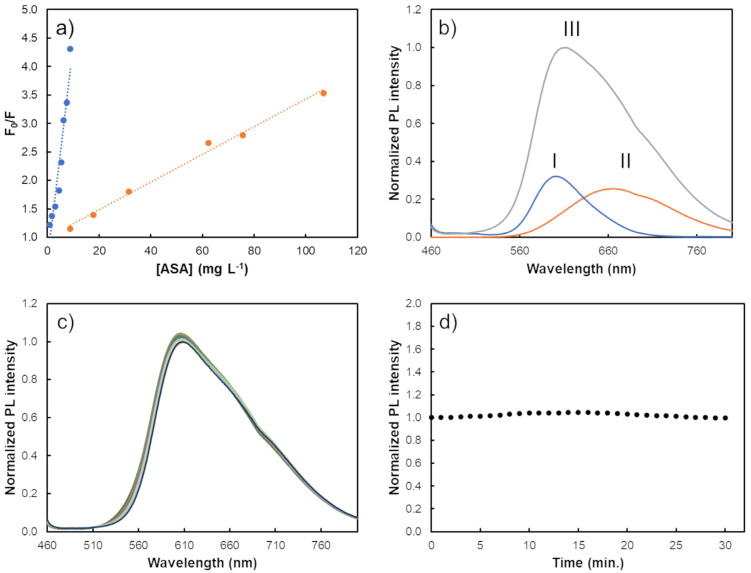
(**a**) Stern–Volmer plots displaying the ratio of the emission intensities for MPA-AIS (orange circles) and MES-CdTe (blue circles) when interacting with increasing concentrations of aspirin. (**b**) PL spectra of (I) MES-CdTe QDs, (II) MPA-AIS QDs and (III) MES-CdTe/MPA-AIS combined probe. (**c**) PL spectra evolution of MES-CdTe/MPA-AIS combined probe and (**d**) the corresponding evolution of the maximum PL intensity during 30 min.

Therefore, a mixture of MES-CdTe (19.00 µg mL^−1^ (*w*/*v*)) and MPA QDs (dilution factor of 1:400 from the crude solution), emitting at 599 and 665 nm, respectively, was evaluated as a multi-emitter nanoprobe for aspirin monitoring. The PL spectrum of the multi-emitter nanoprobe exhibited a very broad band with undefined maximum emission wavelengths due to the overlap of both individual nanoparticle emission bands. This fact also results in a PL intensity enhancement of both emission bands (Figure 7b).

The emission intensity of the combined sensing platform was evaluated for 30 min since the stability of the nanoprobe can affect the reproducibility and accuracy of the PL measurements. Through the analysis of the obtained results (Figure 7c,d), one can conclude that the maximum PL intensity of the combined nanoprobe was very stable throughout the 30 min.

**Figure 8:**
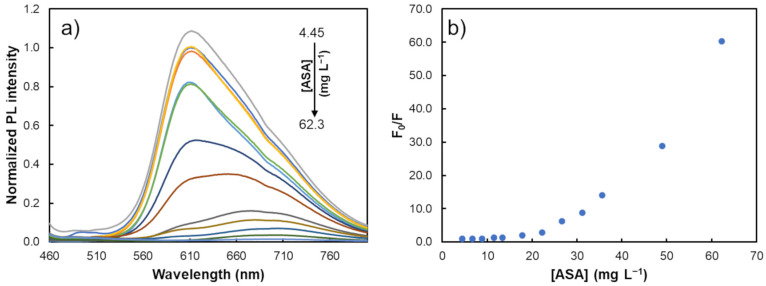
(**a**) PL spectra of the nanohybrid probe in the absence and presence of increasing concentrations of ASA between 4.45 and 62.3 mg L^−1^. (**b**) Stern–Volmer plot fit curves of the interaction between the combined probe and the drug at different concentration levels.

Afterwards, the PL response of the combined nanoprobe was evaluated upon interaction with aspirin at a concentration range of 4.45–62.3 mg L^−1^. The results shown in Figure 8 demonstrated a more evident PL intensity-quenching of the emission band corresponding to the CdTe QDs for ASA concentrations up to 22.25 mg L^−1^. At the same time, a redshift of 42 nm (from 611 to 652 nm) was also observed. Furthermore, for higher ASA concentrations, a PL intensity inhibition of the emission band corresponding to the AIS QDs was also noticed. With this nanohybrid probe composed of CdTe and AIS QDs, the drug showed greater affinity for the MES-CdTe QDs than for the MPA-AIS ones, which is in compliance with the obtained results of the interaction study of the drug with the binary and ternary QDs individually.

### 3.4. Kinetic Determination of ASA

The kinetic behaviour of the PL properties of the combined nanoprobe in the presence and in the absence of ASA was evaluated throughout 30 min. The effect of the drug on the QDs’ PL emissions was noticeable, particularly in the first 10 min, remaining practically unaffected posteriorly (Figure S2—Supplementary Material). Therefore, to ensure a good compromise between reactivity and the sampling rate, the PL analysis time was reduced for 10 min.

#### Optimization and Comparison of Different Chemometric Models

**Figure 9:**
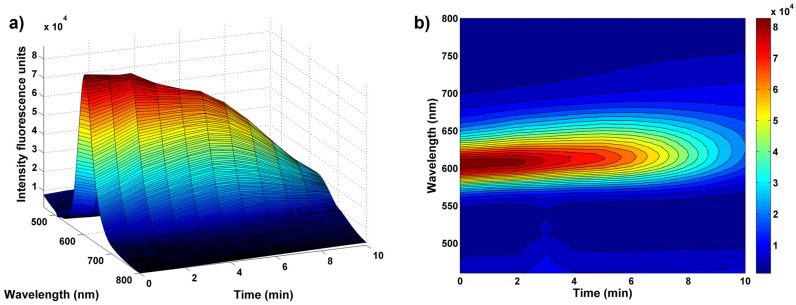
(**a**) Second-order data PL spectra between QDs and ASA standard of 35.6 mg L^−1^, and (**b**) the corresponding plot of the fluorescence emission as function of time and wavelength.

As aforementioned, the obtained second-order data (Figure 9) on this work allow for the quantification of ASA in the presence of uncalibrated species if proper chemometric models are selected. This is possible due to the second-order advantage, as mentioned in the introduction section, achieved through the application of residual bilinearization (RBL) [58]. However, this was not effectively necessary as the other compounds present in the commercial samples, namely caffeine and bisoprolol, did not interact with the PL nanoprobe.

As can be seen, upon the interaction of ASA and the combined nanoprobe, a quenching on the PL signal was observed, as reported before. Four different chemometric models, specifically U-PLS, N-PLS, MLF-NN and RBF-NN, were optimized and compared to identify the most appropriate chemometric tool. This involved testing different pre-processing techniques, as aforementioned. For N-PLS and U-PLS, depending on the pre-processing technique, different numbers of LVs were selected according to the criterion of Haaland and Thomas [46]. For MLF-NN and RBF-NN, depending on the pre-processing technique, different architectures were selected. Then, the validation samples were projected on the best optimized calibration models of each of these chemometric tools to verify their accuracy.

Again, a total of 13 calibration standards within the range of 5.3 to 40 mg L^−1^ were prepared to calibrate and optimise the different chemometric models tested. The obtained results for this optimization are shown in Table 3.

From the analysis of this table, regarding the different pre-processing techniques, it is visible that the best results were obtained with the data just mean-centred. This can be related with the fact that the fluorescence signal is not as prone to artifacts as the signal of near-infrared spectroscopy is; this is evident, for example, in settings where the use of pre-processing techniques to correct scattering effects and temperature variation is crucial, among others. In relation to the different chemometric models tested, namely U-PLS, N-PLS, MLF-NN and RBF-NN, and considering the best calibration model for each of these models (with the fluorescence signal just mean-centred), there are no significant differences among them. However, both the RBF-NN and MLF-NN models yielded better results than U-PLS and N-PLS when applying the second derivative.

The best models obtained for each chemometric tool yielded an R^2^_C_ of 0.98, 0.98, 0.99 and 0.99 U-PLS, N-PLS, MLF-NN and RBF-NN, respectively, and RMSECs of 2.17, 2.17, 1.79 and 1,76 for U-PLS, N-PLS, MLF-NN and RBF-NN, respectively. Moreover, the values predicted in the calibration were all within the confidence intervals (at 95% confidence level) for all the chemometric tools tested. Therefore, the best calibration models demonstrate that the proposed methodology can be a reliable approach for the quantification of ASA.

**Figure 10:**
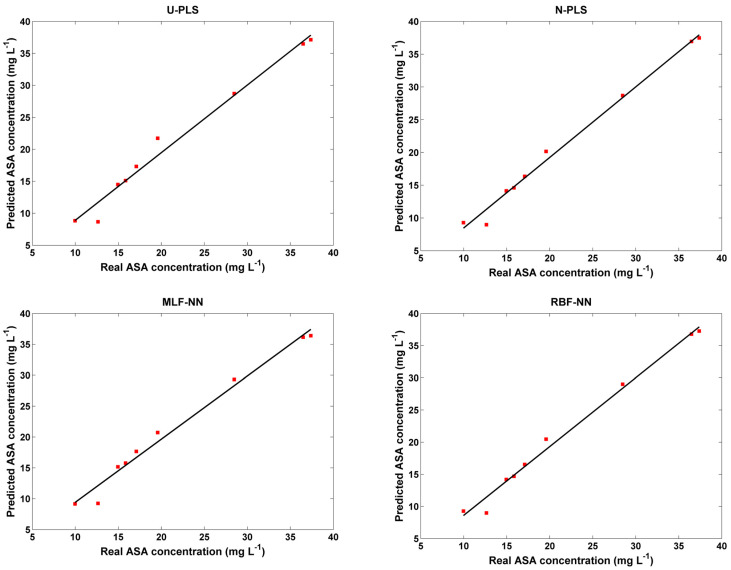
Plot of the predicted ASA concentration values against the real concentration values.

Then, as aforementioned, it is time to perform the evaluation of the accuracy of the best calibration models obtained for each chemometric tool tested. This evaluation was performed using three commercial samples, each one using three different dilutions. The accuracy results are shown in Figure 10 and Table 4.

Analysing the obtained results, it should be highlighted that all the chemometric tools yielded an R^2^_P_ of 0.99 and a RMSEP lower than 2.0 mg L^−1^. Moreover, the obtained REs were lower than 7% for U-PLS and N-PLS and around 10% for the MLF-NN and RBF-NN, confirming the accuracy of all the developed models. This demonstrates the suitability of the hereby proposed methodology in terms of accuracy.

About the different chemometric models tested, it is not possible to indicate the best one as the obtained results were very similar. Nevertheless, it should be mentioned that the U-PLS and N-PLS models need less computation time, are simpler to implement and generate data (such as loadings and regression coefficient vectors) that are easier to interpret than both NNs [43,44].

Globally, the obtained results attest that this approach is capable of quantifying ASA in pharmaceutical samples, even in the presence of uncalibrated species, with a remarkable accuracy and in a simple way.

## 4. Conclusions

The combined nanoprobe involving the mixture of MES-CdTe and MPA-AIS QDs was demonstrated to be a very useful sensing platform for the determination of ASA. The combination of binary and ternary QDs allowed us to obtain a more stable nanoprobe, which was important to perform kinetic measurements. The distinct reactivities of each QD involved in the combined nanoprobe was also important to obtain a specific ASA-response profile, thus facilitating its discrimination when this drug is combined with other APIs. The kinetic measurement of the interaction between the combined nanoprobe and ASA allowed us to acquire three-way array data which make it possible to obtain the second-order advantage. The pairing of the obtained PL kinetic data with the most adequate chemometric models enabled us to accurately quantify ASA, even in the presence of other APIs present in the pharmaceutical formulations. The chemometric tools also allowed the successful use of a combined nanoprobe in which the emission bands of both binary and ternary QDs were overlapped. The chemometric models were able to deal with this issue, demonstrating that they could mathematically decompose the overlapping profile of the emission spectra, therefore performing an accurate determination of the target analyte. This fact demonstrated that by using a chemometric analysis, it is possible to combine several QDs in a multi-emission probe, even when their emission bands are overlapped.

Regarding the pre-processing techniques tested, the obtained results demonstrated that the best results were obtained without the application of any pre-processing technique (just with data mean-centred). As abovementioned, this could rest on the fact that fluorescence data are not prone to signal artifacts as is, for example, the data obtained when using near-infrared spectroscopy (e.g., scattering effects, temperature variation).

Regarding the different chemometric models tested, either in the calibration and in the validation step, there were no significant differences in terms of accuracy. In the calibration step, the only perceptible difference occurred when applying the pre-processing technique using the second derivative, where both the MLF-NN and RBF-NN showed better results than U-PLS and N-PLS. However, there were no significant differences between these chemometric tools when comparing the best approach for each one. Indeed, the best results were attained when the fluorescence signals were just mean-centred, yielding an R^2^_C_ of 0.98, 0.98, 0.99 and 0.99 for U-PLS, N-PLS, MLF-NN and RBF-NN, respectively. In the validation step, there were also no significant differences between the chemometric tools tested. In fact, all the chemometric tools yielded an R^2^_P_ and RMSEP of 0.99 and lower than 2.0 mg L^−1^, respectively, with a RE lower than 7% for U-PLS and N-PLS and around 10% for the MLF-NN and RBF-NN. These results attest the accuracy of all developed models and demonstrate the suitability of the hereby proposed methodology for the quantification of ASA in pharmaceutical samples, showing remarkable accuracy and being performed a simple way.

## Figures and Tables

**Table 1:** PL lifetime values of each synthesized binary CdTe quantum dots.

QDs	Maximum Emission Wavelength (nm)	PL Lifetimes	1st Decay Component	2nd Decay Component	3rd Decay Component	τ_average_ (ns)	QY (%)
TMA-CdTe	605	τ_i_ (ns)	4.6 ± 0.2	23.92 ± 0.09	73.6 ± 0.4	44.4 ± 0.2	17.0 ± 0.2
		B_i_ (%)	6.57	75.65	17.81		
MPA-CdTe	676	τ_I_ (ns)	7.2 ± 0.5	37.0 ± 0.3	80.5 ± 0.5	58.5 ± 0.4	63.1 ± 0.1
		B_i_ (%)	3.17	66.37	30.46		
MES-CdTe	599	τ_I_ (ns)	9.9 ± 0.1	1.94 ± 0.02	40.3 ± 0.5	23.7 ± 0.3	3.1 ± 0.2
		B_i_ (%)	61.76	24.07	14.17		
GSH- CdTe	605	τ_I_ (ns)	37 ± 3	15.5 ± 0.9	68.51 ± 1	54 ± 3	46.0 ± 0.2
		B_i_ (%)	50.69	9.05	40.26		

τi—PL lifetimes of each decay fraction; Bi—fractional intensity; QY—quantum yield.

**Table 2:** PL lifetime values of each synthesized ternary AgInS_2_ quantum dots.

QDs	Maximum Emission Wavelength (nm)	PL Lifetimes	1st Decay Component	2nd Decay Component	3rd Decay Component	τ_average_ (ns)	QY (%)
TMA-AIS	672	τ_i_ (ns)	0.19 ± 0.03	491 ± 2		491 ± 2	9.1 ± 0.1
		B_i_ (%)	10.91	89.09			
TMA-AIS/ZnS	650	τ_I_ (ns)	94 ± 2	465.4 ± 0.8	0.64 ± 0.06	461 ± 1	20.1 ± 0.4
		B_i_ (%)	5.14	94.62	0.25		
MPA-AIS	665	τ_I_ (ns)	130 ± 3	460 ± 1	16.5 ± 0.7	446 ± 2	33.1 ± 0.4
		B_i_ (%)	13.47	85.49	1.04		
D-Pen-AIS/ZnS	603	τ_I_ (ns)	76 ± 1	383.3 ± 0.8	0.92 ± 0.02	376 ± 1	15.1 ± 0.1
		B_i_ (%)	10.45	88.37	1.19		

τi—PL lifetimes of each decay fraction; Bi—fractional intensity; QY—quantum yield.

**Table 3:** Obtained results for the optimization of unfolded-partial least squares (U-PLS), N-way partial least squares (N-PLS), multilayer feed-forward neural networks (MLF-NNs) and radial basis function neural networks (RBF-NNs) models.

Pre-Processing Technique	None *	SNV	SG(15,3,1)	SNV + SG(15,3,1)	SG(7,3,2)	SNV + SG(7,3,2)
U-PLS						
LV	2	1	2	3	1	1
Outliers	---	---	---	1	---	1
RMSEC	2.17	3.91	6.86	2.84	6.86	8.64
R^2^_C_	0.98	0.94	0.80	0.97	0.80	0.66
N-PLS						
LV	1	1	4	3	1	1
Outliers	---	---	---	1	---	1
RMSEC	2.17	3.53	4.22	2.85	6.10	9.05
R^2^_C_	0.98	0.95	0.93	0.97	0.84	0.60
MLF-NN						
Architecture	2-2-1	2-2-1	2-2-1	2-1-1	2-2-1	2-2-1
Outliers	---	---	---	---	---	---
RMSEC	1.79	2.69	3.72	2.60	3.74	2.63
RMSEM	1.08	1.99	2.56	2.74	2.54	2.92
R^2^_C_	0.99	0.97	0.95	0.97	0.95	0.96
RBF-NN						
Architecture	2-2-1	2-3-1	2-3-1	2-2-1	2-2-1	2-3-1
Outliers	---	---	---	1	---	---
RMSEC (mg L^−1^)	1.76	3.42	5.78	2.04	4.88	2.23
R^2^_C_	0.99	0.95	0.86	0.98	0.90	0.98

Legend: * mean-centring; LV—latent variables; SNV—standard normal variate; SG(x,y,z)—Savitzky–Goley (filter points, polynomial order, derivative).

**Table 4:** Predicted results for ASA commercial samples and the respective figures of merit for each chemometric tool.

		U-PLS	N-PLS	MLF-NN	RBF-NN
	Real Values	Predicted Values	Predicted Values	Predicted Values	Predicted Values
	mg L^−1^				
Commercial sample 1	10.0	8.83	9.28	9.27	9.15
	15.8	15.11	14.60	14.69	15.78
	28.5	28.72	28.69	29.00	29.31
Commercial sample 2	12.64	8.67	8.96	8.98	9.24
	17.09	17.31	16.35	16.52	17.67
	36.49	36.49	36.94	36.77	36.15
Commercial sample 3	14.95	14.49	14.13	14.20	15.16
	19.58	21.72	20.16	20.47	20.73
	37.38	37.13	37.47	37.25	36.40
RMSEP (mg L^−1^)		2.03	1.81	1.98	1.85
R^2^_P_		0.99	0.99	0.99	0.99
RE (%)		6.76	6.88	10.38	9.69
LOD (mg L^−1^)		2.82	3.10	3.38	3.26
LOQ (mg L^−1^)		6.76	6.88	10.14	9.79

Legend: RMSEP—root mean square error of prediction; R^2^_P_—coefficient of determination for the prediction; RE—relative percentage error for concentration prediction; LOD—limit-of-determination; LOQ—limit-of-quantification.

## Data Availability

Not applicable.

## Supplementary Materials

The following supporting information can be downloaded at: https://www.mdpi.com/article/10.3390/bios13040437/s1. Figure S1. PL decay curves of (a) Cd-based binary and (b) Cd-free ternary QDs. Figure S2. (a,c) Second-order data PL spectra of combined nanoprobe and (b) the corresponding plot of the fluorescence emission throughout 30 minutes; Second-order data PL spectra between combined nanoprobe and ASA standard of 35.6 mg L^−1^, and (b,d) the corresponding plot of the fluorescence emission as function of time and wavelength.
