# Supplementary material for: Kinetic Determination of Acetylsalicylic Acid Using a CdTe/AgInS2 Photoluminescence Probe and Different Chemometric Models
Source: Biosensors (Basel). 2023 Mar 30;13(4):437. doi: 10.3390/bios13040437 (PMC10135845; doi:10.3390/bios13040437)
Supplement: Supplementary file 1 [file biosensors-13-00437-s001.zip › biosensors-2285972-supplementary.pdf]

biosensors

MDPI

Supplementary Material

Kinetic Determination of Acetylsalicylic Acid Using a CdTe/AgInS2 Photoluminescence Probe and Different Chemometric Models

Rafael C. Castro, Ricardo N. M. J. Páscoa *, M. Lúcia M. F. S. Saraiva, João L. M. Santos and David S. M. Ribeiro *

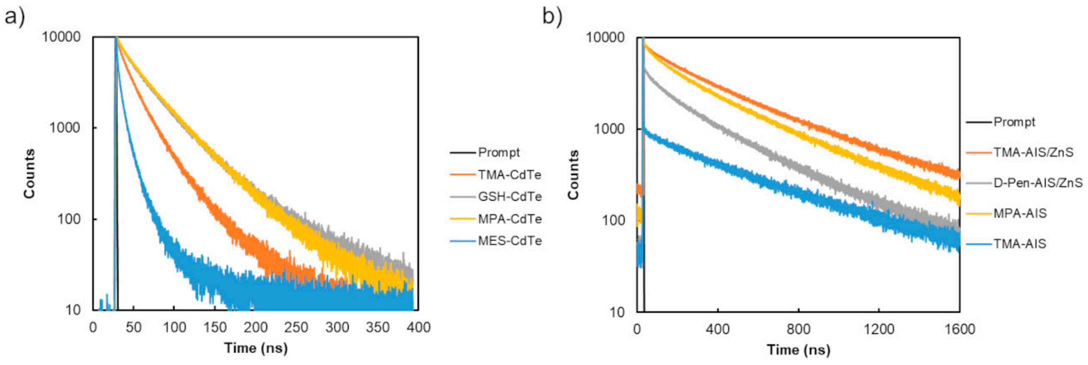

Figure S1. PL decay curves of (a) Cd-based binary and (b) Cd-free ternary QDs.

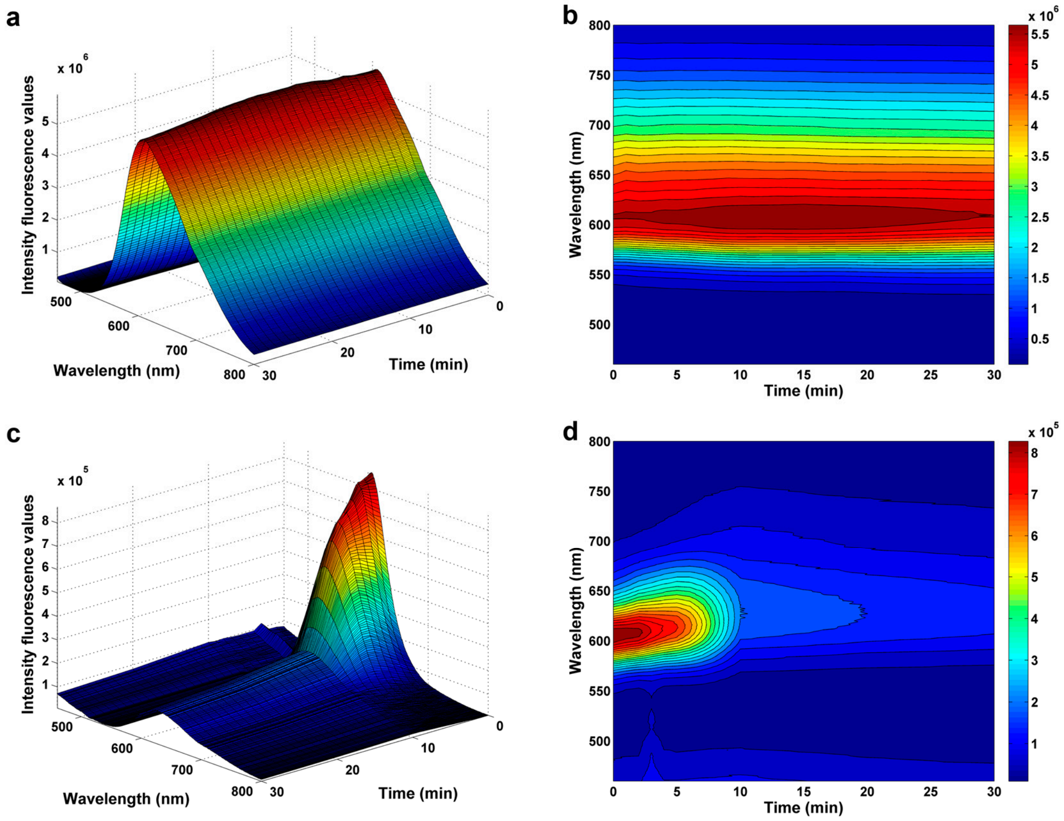

Figure S2. (a,c) Second-order data PL spectra of combined nanoprobe and (b) the corresponding plot of the fluorescence emission throughout 30 minutes; Second-order data PL spectra between combined nanoprobe and ASA standard of 35.6 mg L^{-1}, and (b,d) the corresponding plot of the fluorescence emission as function of time and wavelength.
